# Bilateral anophthalmia in a neonate: a syndromic presentation with multisystem anomalies and diagnostic challenges

**DOI:** 10.1093/omcr/omaf133

**Published:** 2025-08-20

**Authors:** Mira Hallak, Baraa Emran, Fathi Milhem, Abdalfatah Badran, Ajwad Mallak, Orabi Hajjeh, Noor Nabresi

**Affiliations:** Department of Medicine, An Najah National University, Nablus, Palestine; Department of Medicine, An Najah National University, Nablus, Palestine; Department of Medicine, An Najah National University, Nablus, Palestine; Department of Medicine, An Najah National University, Nablus, Palestine; Department of Medicine, An Najah National University, Nablus, Palestine; Department of Medicine, An Najah National University, Nablus, Palestine; Pediatric department, Dr. Thabet Thabet Hospital, Tulkarm, Postal Code P304, Palestine

**Keywords:** anophthalmia, congenital anomaly, syndromic presentation, multisystem anomalies, genetic testing, multidisciplinary care

## Abstract

Anophthalmia is a rare congenital defect where no ocular tissue is seen, and it involves 1 out of 10 000 to 20 000 live births. It is largely part of syndromes and consists of genetic, environmental origins, or multifactorial causes. We present a case of a neonate with bilateral anophthalmia, ambiguous external genitalia, microcephaly, and renal ectopy, suggesting a syndromic etiology. Despite receiving multidisciplinary care, the patient unfortunately succumbed to complications, including refractory respiratory distress and seizures. Early diagnosis relies on timely imaging and confirmation, with management guided by appropriate genetic testing, an approach well illustrated by this case. Anophthalmia presents medical and psychosocial challenges that require a coordinated, multidisciplinary approach.

## Introduction

Anophthalmia is a rare congenital condition characterized by the complete absence of the eye and associated ocular tissue within the orbit, with an estimated incidence of 1 in 10 000 to 20 000 live births, varying by geographic and methodological factors [1]. This condition is classified into two main types: primary anophthalmia, where the eye fails to form during early embryogenesis, and secondary anophthalmia, where initial eye formation occurs but is subsequently disrupted due to external insults such as infections, vascular events, or teratogenic exposures [2]. In primary anophthalmia, the failure of the optic vesicle to develop from the forebrain leads to the absence of key structures such as the optic nerve, optic chiasm, lens, and retina. Secondary anophthalmia is typically attributed to environmental factors that disrupt ocular development during gestation, highlighting the interplay between genetic and external influences [2] [3].

Both types can be unilateral or bilateral, and are frequently associated with systemic syndromes such as CHARGE syndrome, Waardenburg syndrome, Goldenhar syndrome, as well as ocular abnormalities such as microphthalmia and coloboma [4].

Diagnosis often begins with clinical suspicion when a newborn exhibits a small or absent orbit. Imaging techniques, such as computed tomography (CT) and magnetic resonance imaging (MRI), are crucial in confirming the absence of the globe and evaluating associated cranial and orbital abnormalities, such as optic nerve hypoplasia or brain malformations. Additionally, advanced genetic tools such as whole-exome sequencing and chromosomal microarray analysis help uncover genetic mutations and syndromic associations, enabling precise diagnosis and family counseling [5].

Management of anophthalmia involves a multidisciplinary approach to address the medical, functional, and psychosocial needs of the patient. Early socket expansion therapy is essential to promote normal orbital development, followed by prosthetic fitting to enhance cosmetic and psychological outcomes. Genetic counseling plays a critical role in informing families about the etiology, recurrence risks, and potential preventive measures for future pregnancies. Long-term follow-up with specialists, including pediatricians, ophthalmologists, and neurologists, ensures comprehensive care for related abnormalities and developmental support.

## Case presentation

**Figure 1:**
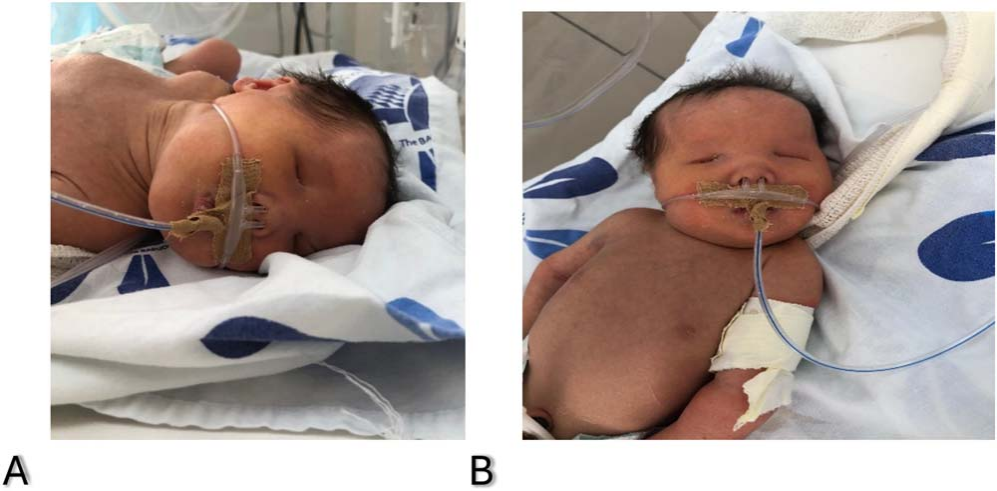
Clinical appearance of the patient with bilateral anophthalmia.

A two-day-old neonate was referred to the hospital following the finding of a bilateral absence of globes during a post-delivery neonatal examination (Fig. 1). A full-term neonate was delivered via scheduled cesarean section to a multigravida mother. The pregnancy was uneventful, and the mother was compliant with her antenatal care visits, during which imaging revealed bilateral anophthalmia. There was no history of drug intake with possible teratogenic effects, smoking, alcohol consumption, or radiation exposure. She did not experience premature rupture of membranes, fever, skin rashes, or other signs suggestive of congenital infection. The older siblings are healthy, with no known intellectual or physical disabilities. However, she had a previous multigestation pregnancy of premature twins, one of whom died shortly after delivery. The father is in good health and without medical comorbidities. The parents are non-consanguineous.

At birth, the patient weighed 1.7 kg, measured 42 cm in length, and had a head circumference of 28 cm. All measurements were below the 3rd percentile, consistent with intrauterine growth restriction (IUGR). Upon delivery, the patient exhibited respiratory distress with a low SpO2 of 70% but was treated accordingly until vital signs stabilized, achieving an SpO2 of 94%.

At the hospital, physical examination revealed a small, hypoactive, jaundiced newborn with no respiratory distress. A thorough systemic examination revealed a small anterior fontanelle, a depressed nasal bridge, ambiguous genitalia, and an anal opening was present but lacked an anal reflex, with a likely fistula through which meconium was being discharged. Ophthalmologic examination confirmed a bilateral absence of globes (anophthalmia) with adhesions at the eyelid margins.

Laboratory evaluation raised concerns for possible sepsis, highlighting elevated inflammatory markers, hyperbilirubinemia, and metabolic acidosis based on arterial blood gas analysis, although clinical signs were absent (Table 1). The newborn was started on a combination of ampicillin and cefotaxime, despite cefotaxime covering a similar spectrum of bacterial pathogens. The addition of ampicillin was considered to ensure broader empirical coverage, particularly for organisms such as *Listeria monocytogenes* and *Group B Streptococcus*, which may be more susceptible to ampicillin in neonates. This was alongside sodium bicarbonate and supportive care, including nasogastric feeding and intravenous fluids. A follow-up evaluation three days later revealed resolving jaundice and normalization of laboratory results.

**Table 1:** Laboratory findings of the neonate upon hospital admission.

**Lab**	**Result**	**Unit**	**Reference Range (Neonate)**
**White Blood Cell Count**	**32 200**	**/mm** ^**3**^	**9000–30 000**
**C-Reactive Protein (CRP)**	**3**	**mg/L**	**<1**
**Total Serum Bilirubin**	**7.3**	**mg/dL**	**1–12 (depending on age in hours/days)**
**Arterial Blood Gas (ABG):**			
**pH**	**7.18**	**–**	**7.35–7.45**
**HCO3**	**14.3**	**mEq/L**	**20–26**
**PCO2**	**37.8**	**mmHg**	**35–45**

Brain ultrasound showed no evidence of intraventricular hemorrhage, ventricular dilation, or midline shift. Abdominal ultrasound revealed the absence of a gallbladder. Both kidneys were normal in size and shape, though the left kidney was located in the mid-abdomen adjacent to the right kidney.

Ophthalmological consultation confirmed the diagnosis and recommended the insertion of an eye conformer or gel ball within the upcoming months. MRI and genetic testing, including karyotype analysis, were planned to further evaluate the condition. However, the patient unfortunately developed respiratory distress with recurrent convulsions several days into admission, eventually leading to death.

## Discussion

Anophthalmia is considered a disabling, congenital eye anomaly characterized by the complete absence of ocular tissue. This anomaly can present as either unilateral or bilateral [6]. Anophthalmia results from disruptions during early embryogenesis, around 3–4 weeks of gestation, particularly affecting optic vesicle formation [7]. ‘Clinical anophthalmia’ refers to the absence of visible ocular tissue, although small residual eye structures may still be identified on histological examination or neuroimaging [8]. Anophthalmia lies within a phenotypic continuum alongside microphthalmia and coloboma, collectively known as MAC. Microphthalmia denotes a reduced eye size with small axial length, often due to impaired ocular growth, whereas coloboma is defined by defects in the iris, retina, and optic nerve due to insufficient closure of the orbital fissure [9]. Advanced imaging techniques are crucial for diagnosing anophthalmia, with MRI being especially valuable. MRI can confirm the absence of ocular tissue within the orbit while occasionally identifying soft, amorphous remnants. This is particularly important in distinguishing clinical anophthalmia from severe microphthalmia, as MRI provides detailed visualization of orbital structures and associated abnormalities [10].

Anophthalmia may result from multifactorial causes, including environmental teratogens, genetic mutations, chromosomal abnormalities, or congenital infections like rubella and toxoplasmosis [11]. Key genetic contributors to bilateral anophthalmia include *de novo*, heterozygous, loss-of-function mutations in SOX2 or OTX2, which regulate crucial eye development pathways such as optic vesicle formation and forebrain specification [1, 8]. Other mutations, such as those in PAX6, BMP4, and FOXE3, have been linked to syndromic or familial cases, particularly in consanguineous populations [12]. Advances in diagnostic tools, including next-generation sequencing and array comparative genomic hybridization (aCGH), have significantly improved the identification of genetic etiologies [7]. These advancements will aid in understanding genotype–phenotype correlations, improve prognostic insights, and guide personalized care strategies.

Anophthalmia can occur either as an isolated condition due to developmental genetic mutations or as part of a syndromic presentation associated with systemic conditions such as CHARGE syndrome, oculo-auriculo-vertebral spectrum (Goldenhar syndrome), Fraser syndrome, VACTERL association, and Waardenburg syndrome [13]. The clinical features of these systemic conditions are provided in Table 2. Maternal factors like vitamin A deficiency, smoking, and drug use during pregnancy contribute significantly to its etiology. Additionally, advanced maternal age and subfertility are notable risk factors, likely linked to chromosomal abnormalities and altered embryonic development [9].

**Table 2:** Systemic conditions associated with anophthalmia.

**Syndrome**	**Clinical features**	**References**
CHARGE syndrome	Coloboma or anophthalmia of the eye; heart defects; atresia choanae; retardation of the mental and somatic development; genital abnormalities; ear anomalies.	Taha Najim R, et al.
Oculo-auriculo-vertebral spectrum (Goldenhar syndrome)	Craniofacial anomalies (anophthalmia, microphthalmia, epibulbar dermoids), auricular malformations, vertebral defects, and occasionally systemic involvement of the heart, kidneys, or gastrointestinal tract.	Taha Najim R, et al.
Fraser syndrome	Cryptophthalmos (fusion of the eyelids), syndactyly, renal agenesis, and urogenital anomalies.	Stoll C, et al.
VACTERL association	Vertebral defects, anal atresia, cardiac anomalies, tracheoesophageal fistula, renal abnormalities, and limb malformations, with potential secondary eye involvement.	Stoll C, et al.
Waardenburg syndrome	Hearing loss, eye abnormalities including microphthalmia or anophthalmia, and distinctive facial features.	Ragge N, et al.

The findings in our case strongly suggest a syndromic etiology, as the patient presents with bilateral anophthalmia along with multiple anomalies, including microcephaly, ambiguous genitalia, and an ectopic kidney. These features align with conditions such as CHARGE and Fraser syndrome, which are known to be associated with anophthalmia and systemic anomalies. CHARGE syndrome is characterized by a combination of ocular, ear, and other systemic defects, while Fraser syndrome often presents with similar developmental issues. These syndromes provide a framework for understanding the complex presentation in our patient. To further clarify the underlying cause, genetic testing, including karyotype analysis, is essential. Additionally, genetic counseling for the parents should be strongly recommended to assess recurrence risks, provide family planning options, and offer psychosocial support.

## Conclusion

Anophthalmia is a rare congenital anomaly with significant medical, genetic, and psychosocial implications. We present a case that reflects the need to diagnose anophthalmia as part of a wide spectrum of syndromic conditions, when presented together with associated abnormalities such as ambiguous genitalia and ectopic position of the kidneys. Further imaging and genetic testing have become imperative for differential diagnosis from similar conditions and determining a multifactorial etiology. Multidisciplinary care remains the cornerstone, which attends to the medical, functional, and psychological needs of affected individuals and provides necessary genetic counseling to the family. Continued advances in diagnostic tools and therapeutic strategies are essential to improving outcomes for affected individuals.

## References

[1] Skalicky SE, White AJ, Grigg JR et al. Microphthalmia, anophthalmia, and coloboma and associated ocular and systemic features: understanding the spectrum. Ophthalmology 2013;131:1517–24. 10.1001/jamaophthalmol.2013.5305 24177921

[2] Källén B, Robert E, Harris J. The descriptive epidemiology of anophthalmia and microphthalmia. Int J Epidemiol 1996;25:1009–16. 10.1093/ije/25.5.1009 8921488

[3] Dedushi K, Hyseni F, Musa J et al. A rare case of anophthalmia without any family history and antenatal risk factors. Cureus 2021;16:3772–5. 10.1016/j.radcr.2021.09.001 PMC8493492 34630815

[4] Ragge N, Subak-Sharpe I, Collin J. A practical guide to the management of anophthalmia and microphthalmia. Eye (Lond) 2007;21:1290–300. 10.1038/sj.eye.6702858 17914432

[5] Slavotinek AM, Garcia ST, Chandratillake G et al. Exome sequencing in 32 patients with anophthalmia/microphthalmia and developmental eye defects. Am J Med Genet A 2015;167A:468–73. 10.1111/cge.12543 PMC4452457 25457163

[6] Verma AS, FitzPatrick DR. Anophthalmia and microphthalmia. Orphanet J Rare Dis 2007;2:47. 10.1186/1750-1172-2-47 18039390 PMC2246098

[7] Harding P, Brooks BP, FitzPatrick D et al. Anophthalmia including next-generation sequencing-based approaches. Eur J Hum Genet 2020;28:388–98. 10.1038/s41431-019-0479-1 31358957 PMC7029013

[8] Harding P, Moosajee M. The molecular basis of human anophthalmia and microphthalmia. J Dev Biol 2019;7:16. 10.3390/jdb7030016 31416264 PMC6787759

[9] Källén B, Tornqvist K. The epidemiology of anophthalmia and microphthalmia in Sweden. Eur J Epidemiol 2005;20:345–50. 10.1007/s10654-004-6880-1 15971507

[10] Celebi ARC, Sasani H. Differentiation of true anophthalmia from clinical anophthalmia using neuroradiological imaging. World J Radiol 2014;6:515–8. 10.4329/wjr.v6.i7.515 25071894 PMC4109105

[11] Taha Najim R, Topa A, Jugård Y et al. Children and young adults with anophthalmia and microphthalmia: diagnosis and management. Acta Ophthalmol 2020;98:848–58. 10.1111/aos.14427 32436650

[12] Williamson KA, FitzPatrick DR. The genetic architecture of microphthalmia, anophthalmia and coloboma. Eur J Med Genet 2014;57:369–80. 10.1016/j.ejmg.2014.05.002 24859618

[13] Stoll C, Dott B, Alembik Y et al. Associated anomalies in anophthalmia and microphthalmia. Eur J Med Genet 2024;67:104892. 10.1016/j.ejmg.2023.104892 38110175

